# Pre-sintered Y-TZP sandblasting: effect on surface roughness, phase transformation, and Y-TZP/veneer bond strength

**DOI:** 10.1590/1678-7757-2017-0131

**Published:** 2017

**Authors:** Carla Müller Ramos-Tonello, Bruno Freitas Trevizo, Raphaela Farias Rodrigues, Ana Paula Rodrigues Magalhães, Adilson Yoshio Furuse, Paulo Noronha Lisboa, Americo Sheitiro Tabata, Ana Flávia Sanches Borges

**Affiliations:** 1 Faculdades Metropolitanas Unidas, Faculdade de Odontologia, São Paulo, SP, Brasil; 2 Universidade de São Paulo, Faculdade de Odontologia de Bauru, Bauru, SP, Brasil; 3 Universidade de São Paulo, Faculdade de Odontologia de Bauru, Departamento de Dentística, Endodontia e Materiais Dentários, Bauru, SP, Brasil; 4 Univ. Estadual Paulista, Faculdade de Ciências, Departamento de Física, Bauru, SP, Brasil

**Keywords:** Zirconium, Ceramics, Surface properties, Shear strength

## Abstract

Sandblasting is a common method to try to improve the Y-TZP/veneer bond strength of dental prostheses, however, it may put stress on zirconia surfaces and could accelerate the t→m phase transformation. Y-TZP sandblasting before sintering could be an alternative to improve surface roughness and bonding strength of veneering ceramic.

**Objectives.:**

The aim of this study was to analyze the effect of Y-TZP pre-sintering sandblasting on surface roughness, phase transformation, and the Y-TZP/veneer shear bond strength.

**Material and Methods.:**

The Y-TZP specimen surface underwent sandblasting with aluminum oxide (50 μm) pre-sintering (Z-PRE) and post-sintering (Z-POS). Z-CTR was not subjected to surface treatment. After ceramic veneer application, the specimens were subjected to shear bond testing. Surface roughness was analyzed by confocal microscopy. Y-TZP monoclinic and tetragonal phases were evaluated by micro-Raman spectroscopy. Shear bond strength and surface roughness data were analyzed by One-way ANOVA and Tukey tests (α=0.05). Differences in the wave numbers and the broadening bands of the Raman spectra were compared among groups.

**Results.:**

Z-POS (9.73±5.36 MPa) and Z-PRE (7.94±2.52 MPa) showed the highest bond strength, significantly higher than that of Z-CTR (5.54±2.14 MPa). The Ra of Z-PRE (1.59±0.23 µm) was much greater and significantly different from that of Z-CTR (0.29±0.05 µm) and Z-POS (0.77±0.13 µm). All groups showed bands typical of the tetragonal (T) and monoclinic (M) phases. Y-TZP sandblasting before sintering resulted in rougher surfaces but did not increase the shear bond strength compared to post-sintering and increased surface defects.

**Conclusions.:**

Surface treatment with Al_3_O_2_, regardless of the moment and application, improves the results of Y-TZP/veneer bonding and is not a specific cause of t→m transformation.

## Introduction

The employment of yttria-stabilized tetragonal zirconia (Y-TZP) by computer-aided design/computeraided manufacturing (CAD-CAM) systems is an accomplished approach to reduce the number of steps in prosthetic manufacturing. Moreover, Y-TZP presents properties such as fracture toughness^6^, strength^12^ ^,^ ^22^, and biocompatibility^33^, which allow it to be employed as a substitute for metal-support fixed dental prosthesis. Ceramic veneers are applied to Y-TZP for esthetic reasons, and their effective bonding is needed for the long-term performance of all ceramic restoration^18^ ^,^ ^35^.

A zirconia-feldspathic veneer has a 13% to 15% rate of failure for up to 5 years^26^. This clinical failure may be associated with chipping, cohesive within the feldspathic layer, or by delamination with adhesive failure at the zirconia-veneer interface^1^ ^,^ ^10^. The differences in coefficients of thermal expansion of each ceramic^12^, the resultant stress of temperature that varies at the Y-TZP/veneer interface^2^ ^,^ ^13^, and poor thermal diffusivity^4^ are factors, either isolated or in conjunction, that may be responsible for delamination (adhesive failures), which is the most common failure^1^. Even veneer cohesive failures (chipping) occur on fragile components of the set. The origin of this type of failure is the most tensile area of the interface between two ceramics^19^. Therefore, improving the bond between the veneer and the zirconia leads to avoidance of interface failures.

The maneuvers to increase surface roughness in an attempt to improve the Y-TZP/veneer bond strength are not always satisfactory due to Y-TZP polycrystalline microstructure and physical properties^32^. The use of hydrofluoric acid does not imply surface roughness for mechanical retention^32^. Therefore, more aggressive mechanical abrasion methods are required, possibly creating surface flaws and reducing the strength of the material^16^ ^,^ ^21^.

Different surface treatments on Y-TZP were evaluated, mostly on the surface of post-sintered zirconia, such as sandblasting, mechanical grinding, silica coating, plasma spray treatment, liner, and laser-etching^7^ ^,^ ^9^ ^,^ ^17^ ^,^ ^18^ ^,^ ^27^. Sandblasting is a useful method, however, it may put stress on zirconia surfaces and accelerate tetragonal-to-monoclinic (t→m) phase transformation^20^. A recent study^14^ shows that sandblasting before (and not after) Y-TZP sintering improves surface roughness by over 500% and could improve the bonding strength of veneering ceramic. Considering that the post-sintered surface treatments weaken the structure of zirconia, increasing the risk of fracture and zirconia damage, the use of pre-sintering surface treatment could be an important way to increase the strength of the zirconia-veneer interface.

The aim of this study, therefore, was to analyze the effect of Y-TZP pre-sintering sandblasting on surface roughness, phase transformation, and Y-TZP/veneer shear bond strength.

## Material and methods

### Specimen preparation

**Figure 1:**
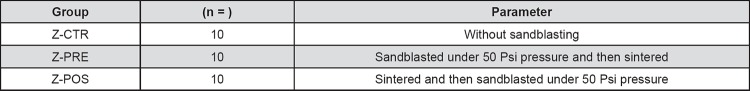
Surface treatments for Y-TZP

Pre-sintered zirconia blocks (IPS e.max Zircad, Ivoclar Vivadent AG), which consisted of 95% ZrO_2_ and 5% HfO_2_+Al_2_O_3_+Y_2_O_3_+Others, were cut with a diamond saw (Isomet 4000, Buehler) into 15 mm diameter and 2.4 mm thickness under water (n=10), and then the surface of each disk was polished with waterproof silicon-carbide paper until reaching 1,000#. The specimens were randomly divided into three groups (Figure 1) according to the surface treatment. Groups Z-PRE (sandblasted before sintering) and Z-POS (sandblasted after sintering) were sandblasted with 50 μm aluminum oxide particles under 50 Psi pressure for 10 s from a distance of 15 mm^25^ by a sandblasting instrument (Trijato, Essence Dental). All the Y-TZP specimens were sintered in a programmable furnace (Infire HTC, Sirona, Dental GmbH) at a cycle of 7 h and 52 min (12°C *per* min until 1500°C; 120 min in 1500°C; cooling at 12°C *per* min; and 1 h and 27 min of holding time) according to the manufacturer's sintering specifications.

**Figure 2:**
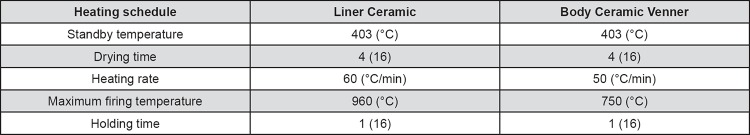
Heating condition for IPS e.max Ceramic Veneer application recommended by the manufacturer (Ivoclar Vivadent AG, Schaan, Liechtenstein)

Specimens of each group were veneered with Ceramic Veneer IPS e.max Ceram (Ivoclar Vivadent AG) for shear bond testing. According to the manufacturer's instructions, a thin layer (0.1 mm) of liner ceramic (Zirliner, Ivoclar Vivadent AG) was applied on all the Y-TZP disks after firing (EDG Equipamentos) at 900°C for 30 min. A custom-designed metallic device^23^ was employed for the standardized application of the ceramic veneer (5 mm diameter and 5 mm thickness). The specimens were sintered at 820°C for 30 min (Figure 2) and placed in a PVC cylinder of 10 mm in diameter with acrylic resin (JET, Classico).

### Shear Bond Test

**Figure 3:**
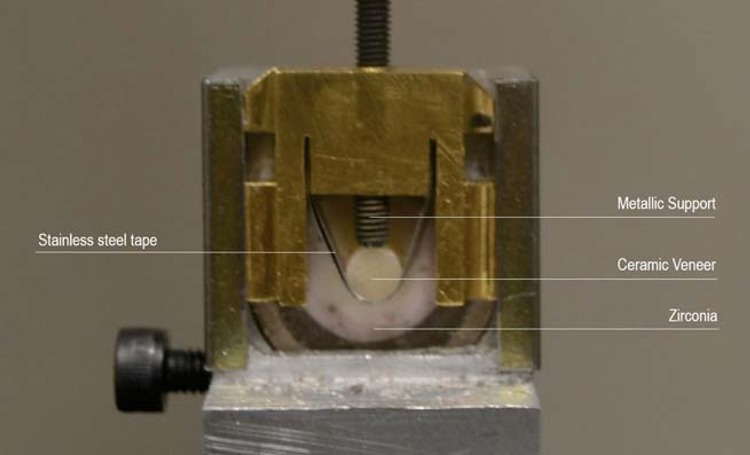
Custom-designed metallic device for shear bond testing with stainless steel tape and metallic support to avoid bending stress during the test

Specimens (n = 10) were subjected to a shear test with a universal testing machine with a load cell of 50 Kgf and a mechanical testing device with stainless steel tape that provided sliding between the two tested surfaces30 at a speed of 1 mm/min until fracture. This device was developed by Ramos, et al.^23^ (2014) to minimize bending stress. For that, stainless steel tape produced smaller tensile and compression forces on the interface, as described by Sinhoreti, et al.^30^ (2001) than those obtained from other tests using a chisel and orthodontic wires. Moreover, the support was adapted to the upper face of the stainless steel tape to minimize any possible bending stresses and cleavage^23^ (Figure 3).

### Failure mode analysis

The analysis of the Y-TZP/veneer interface was performed for all specimens with optical microscopy (Discovery V8 Stereo, Carl Zeiss Microimaging GmbH) at 32x magnification. A failure between the zirconia and ceramic veneer was defined as "adhesive", while a failure within either the zirconia or ceramic veneer was defined as "cohesive". The term "mixed" failure was employed to describe the combination of these two types of failure.

### Surface roughness

Surface roughness (n = 3) was measured using confocal microscopy (Leica Microsystems, Wetzlar). For each Y-TZP specimen, surface roughness was measured at three different locations, then these values were averaged to obtain the average surface roughness (Ra).

The 3D roughness of one specimen in each group was also analyzed by scanning electron microscopy.

### Phase analysis

Y-TZP specimens (n=3) were evaluated by micro-Raman spectroscopy (Jobin Yvon Raman Micro, model T64000, Groupe Horiba) to identify the typical bands of the crystalline phase (monoclinic and tetragonal)^24^. For this, argon laser scanning (SpectraPhysic, Inc.) was performed for excitation at 514.5 nm radiation and was kept at 10 mW to avoid any thermal damage. With microscope magnification of 500x, the beam was focused and scanning was performed in five regions.

The spectra of each specimen were recorded with a CCD camera (Spectra Group One-Horiba) after analysis of the double monochromator with a focal length of 0.64 mm and diffraction grating (1800 grooves/mm)^24^.

### Statistical analysis

Shear bond strength and surface roughness data were analyzed by One-way ANOVA and pairwise multiple comparison Tukey test (α=0.05). The failure types were classified with optical microscopy and calculated as a percentage for each group. Differences in the wave numbers and the broadening bands of the Raman spectra were compared among groups.

## Results

### Shear Bond Test

**Figure 4:**
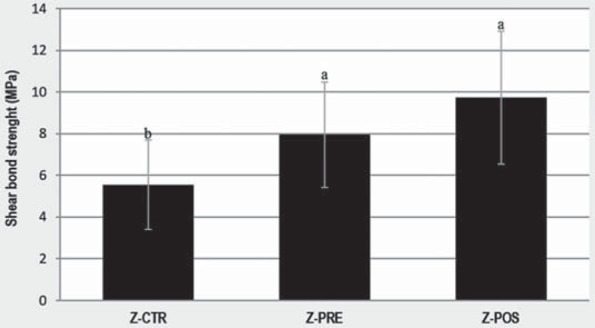
Shear bond strength of each group. Vertical bars indicate the standard deviation and similar letters indicate non-significant differences (p>0.05)

Figure 4 shows the results of the shear bond strength test. Z-PRE (7.94±2.52 MPa) and Z-POS (9.73±5.36 MPa) showed the highest strength, significantly higher than that of Z-CTR (5.54±2.14 MPa), but not significantly different from each other.

### Failure mode analysis

**Figure 5:**
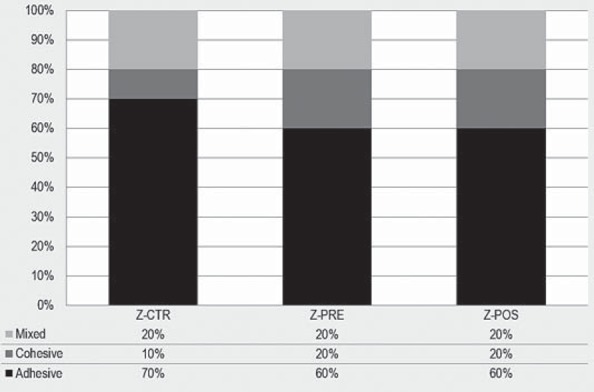
Percentage of failure types

**Figure 6:**
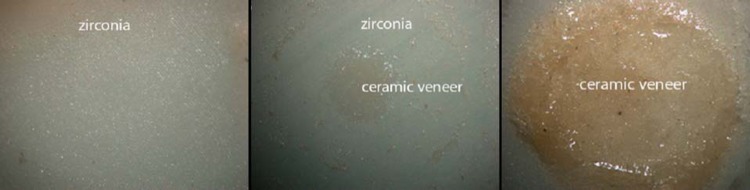
Failure types: adhesive failure (zirconia), mixed failure (zirconia and ceramic veneer), and cohesive failure (ceramic veneer)

Failure mode and distribution for each group are presented in Figures 5 and 6. All groups showed adhesive type failures, especially Z-PRE and Z-POS, which showed 60% adhesive failures. Few cohesive failures were observed, and none of the specimens were fractured within the zirconia.

### Surface roughness

**Figure 7:**
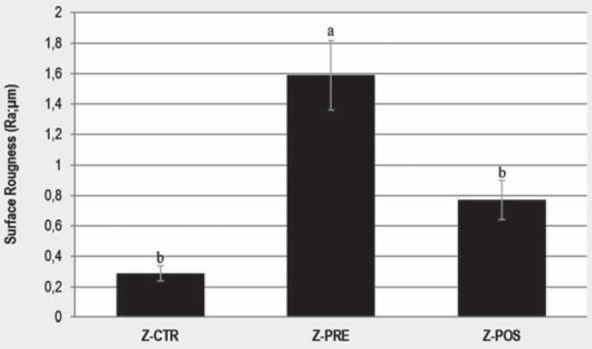
Surface roughness after surface treatment. Vertical bars indicate standard deviation and similar letters indicate non-significant differences (p>0.05)

**Figure 8:**
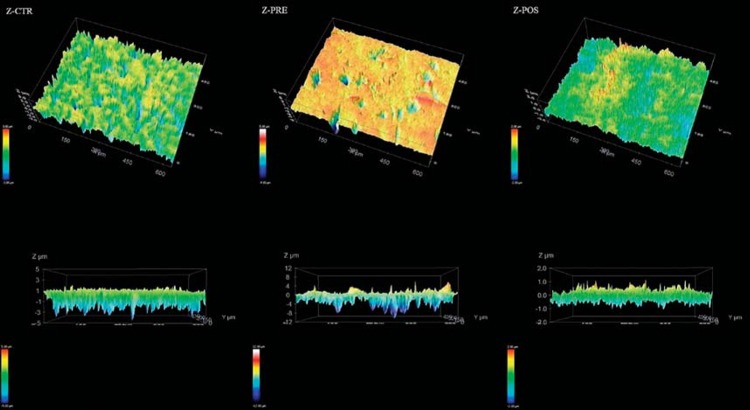
3D representative images of view of surface. Z-CTR – prominent deep valleys (blue sites) and crests distributed homogeneously over the surface. In the deep view of the surface, the values are under -5 µm, and there are slight crests along the surface. Z-POS – deep valleys (blue sites) and crests (red sites) concentrated in certain areas of the surface, with more heterogeneous distribution, ranging from 1 µm to -1 µm. Z-PRE – absence of crests over the surface and areas of localized and prominent deep valleys under -12 µm in size

**Figure 9:**
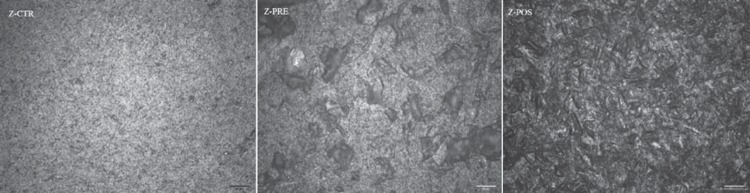
Surface image at 20 × magnification. Z-CTR – homogenous surface with no evident visible signs of damage. Z-POS – surface pattern after post-sintering treatment showing irregularities along the surface. Z-PRE – areas of localized irregularities along the surface after pre-sintering treatment

The comparison of surface roughness is shown in Figure 7. A comparison of the average surface roughness (Ra) for Z-CTR (0.29±0.05 µm), Z-PRE (1.59±0.23 µm), and Z-POS (0.77±0.13 µm) shows that the Ra of Z-PRE is much greater than and significantly different from that of Z-CTR and Z-POS (*p*<0.001). Z-CTR and Z-POS Ra values are not significantly different. The reconstructed images of 3D roughness representative of each group are shown in Figure 8, and surface images at 20x magnification are shown in Figure 9. Z-PRE showed more irregular profiles.

### Phase analysis

**Figure 10:**
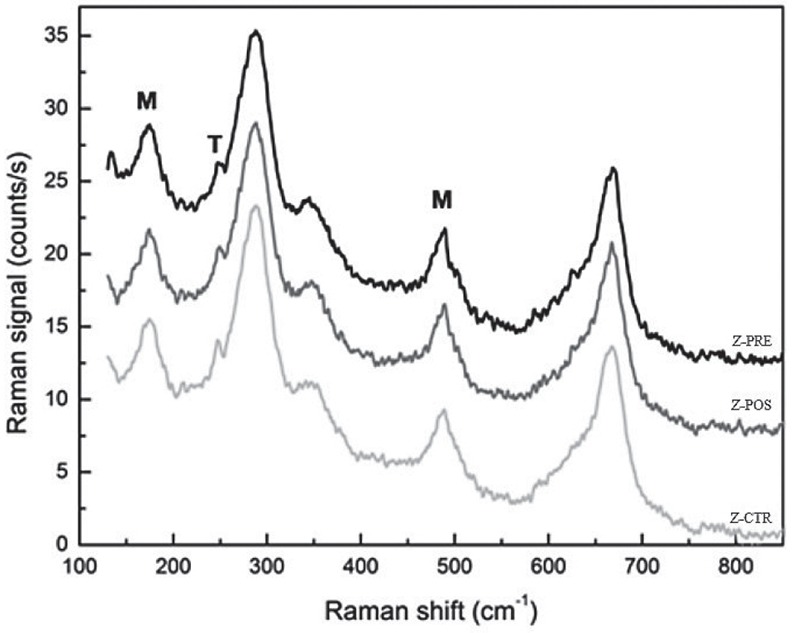
Raman strong peaks for monoclinic (M) and tetragonal (T) phases

All groups showed bands typical of the tetragonal (T) and monoclinic (M) phases. The most prominent peaks found related to monoclinic phases were approximately ~178 and ~474cm^-1^, while those related to tetragonal phases were ~142 and ~256 (Figure 10).

## Discussion

Surface treatments have been recommended to improve the Y-TZP/veneer bond strength by micromechanical interlocking^7^ ^,^ ^9^, even knowing that other factors can influence bond strength, such as the sintering cycle of veneer, cooling rate, and thermal variations^18^ ^,^ ^23^. The sandblasting method was expected to increase surface roughness. Sandblasting at different moments (pre- or post-sintering) resulted in different roughness of Y-TZP^14^. The roughness of Z-PRE was significantly higher than that of Z-POS and Z-CTR. These results could be explained by the much lower hardness of Y-TZP before being sintered, which resulted in a rougher zirconia surface through sandblasting and thus a larger surface area available for mechanical interlocking^14^.

The approach of performing pre-sintering surface treatment is justified as it improves the shear bond strength between Y-TZP and veneer, when compared to an untreated surface^18^. In this study, sandblasting before sintering (Z-PRE) resulted in similar shear bond strength to after sintering (Z-POS), but significantly greater strength than the untreated surface method (Z-CTR). One of the limitations of the current study is that it did not consider the aging of the specimens. Polycrystalline ceramics under wet and cyclic loading conditions are most susceptible to subcritical crack growth^28^. Nevertheless, thermocycling does not necessarily make a difference in bond strength results^23^.

The higher roughness obtained by pretreatment did not result in more bond strength. Thus, the surface roughness/shear bond strength relation may not be linear^19^ and the excessive rough surface may lead to stress concentration, which could consequently weaken the interfacial bonding^15^. Considering that more adhesive failures were found for all studied groups (Figure 4), which is expected for shear bond tests^30^, the device with support adapted to the upper face^23^ was effective in tension distribution along the Y-TZP/veneer interface^3^.

The presence of surface irregularities generated in pretreatment (Figure 9C) indicates that the use of mechanical abrasion in pre-sintering methods can be more aggressive, leading to surface flaws, microfractures that would reduce functional strength, and premature and catastrophic failure of material^21^ ^,^ ^35^. Porosity has a negative effect on the cohesive strength of the materials by reducing the pore cross-section area, through which a load is applied, acting as a stress concentrator^5^. The untreated surface shows no visible evidence of irregularities (Figure 9A). Sandblasting modifies the surface, as seen in Figure 8, and increases the shear bond strength, regardless of treatment time.

Sandblasting is a useful tool to improve retention, however, it may put stress on zirconia surfaces and accelerate the t→m phase transformation^20^ ^,^ ^31^. Hydrothermal or mechanical stress may be responsible for phase transformation^11^. Moreover, the contact with the veneering ceramic may be the cause of the important phase transformation at the interface between zirconia and veneer due to an influence of the amount of moisture present in the veneering porcelain on the faceting grain of zirconia^31^.

The effects of t→m phase transformation are governed by the transformation toughening mechanism that occurs at superficial grains on the ceramic surface, leading to volume increase (~3 to 5%) at a localized area around the superficial defects^28^. After that, t→m phase transformation spreads throughout the material subsurface, resulting in grain pullout and an increase in roughness^29^, jeopardizing the strength, fracture toughness, and density of Y-TZP structures^8^, which is known as low-temperature degradation^8^ ^,^ ^28^.

Micro-Raman spectra of Y-TZP contain important information that can be obtained from the band positions, their intensities, and shapes^34^. The micro-Raman analysis to detect crystalline phases in polycrystalline ceramics is a very useful tool^24^. Even peaks are distinguished in the tetragonal phase (~142, ~256, ~320, ~466, and ~637 cm^–1^), and the most characteristic are a sharp band at 142 cm^–1^ and a broader band at 256 cm^–1^. For the monoclinic phase (~178, ~190, ~219, ~303, ~331, ~345, ~379, ~474, ~500, ~534, ~559, ~615, and ~638 cm^–1^), there is a notable doublet at 178 and 190 cm^–1^ ^34^. Peaks of tetragonal (~142; ~256) and monoclinic (~178; ~474) crystal structures were identified in all studied groups. Pre-sintering sandblasting presented the same Raman bands as post-sintered and untreated Y-TZP surfaces, even with the differences in roughness.

## Conclusions

Within this limited study, the experimental results show that Y-TZP sandblasting before sintering resulted in rougher surfaces, but did not increase the shear bond strength compared to post-sintering treatment and increased surface defects. The surface treatment with Al_3_O_2_, regardless of the moment and application, improved the results of Y-TZP/veneer bonding. The tested approaches for surface treatment did not result in different metastability of tetragonal zirconia. The long-term consequence of the pre-sintered treatment on the zirconia-veneer interface needs to be further investigated.
